# Lifestyles and Risk Factors Associated with Adherence to the Mediterranean Diet: A Baseline Assessment of the PREDIMED Trial

**DOI:** 10.1371/journal.pone.0060166

**Published:** 2013-04-29

**Authors:** Emily A. Hu, Estefania Toledo, Javier Diez-Espino, Ramon Estruch, Dolores Corella, Jordi Salas-Salvado, Ernest Vinyoles, Enrique Gomez-Gracia, Fernando Aros, Miquel Fiol, Jose Lapetra, Lluis Serra-Majem, Xavier Pintó, Maria Puy Portillo, Rosa M. Lamuela-Raventos, Emilio Ros, Jose V. Sorli, Miguel A. Martinez-Gonzalez

**Affiliations:** 1 Department of Nutrition, Harvard School of Public Health, Harvard University, Boston, Massachusetts, United States of America; 2 Department of Preventive Medicine and Public Health, School of Medicine-Clinic, University of Navarra, Pamplona, Navarra, Spain; 3 RETIC RD06/0045, Instituto de Salud Carlos III (ISCIII), Spanish Government, Madrid, Spain; 4 CIBER Fisiopatología de la Obesidad y Nutrición (CIBER obn), Instituto de Salud Carlos III (ISCIII), Spanish Government, Madrid, Spain; 5 Primary Health Care Center of Tafalla-Osasunbidea, Tafalla, Navarra, Spain; 6 Department of Internal Medicine, Institut d’Investigacions Biomediques August Pi Sunyer (IDIBAPS), Hospital Clinic, University of Barcelona, Barcelona, Spain; 7 Department of Preventive Medicine, University of Valencia, Valencia, Spain; 8 Human Nutrition Department, IISPV, Universitat Rovira i Virgili, Reus, Spain; 9 La Mina Primary Care Center, University of Barcelona, Spain; 10 IDIAP JordiGol. Barcelona, Spain; 11 Department of Preventive Medicine, University of Malaga, Malaga, Spain; 12 Department of Cardiology, University Hospital of Alava, Vitoria, Spain; 13 Institute of Health Sciences IUNICS, University of Balearic Islands, and Hospital Son Espases, Palma de Mallorca, Spain; 14 Department of Family Medicine, Primary Care Division of Sevilla, San Pablo Health Center, Sevilla, Spain; 15 Department of Clinical Sciences, University of Las Palmas de Gran Canaria, Las Palmas, Spain; 16 Lipids and Vascular Risk Unit, Internal Medicine, Hospital Universitario de Bellvitge, Hospitalet de Llobregat, Barcelona, Spain; 17 Department of Nutrition and Food Science, School of Pharmacy, University of the Basque County (UPV/EHU), Vitoria, Spain; 18 Department of Nutrition and Food Science, School of Pharmacy, XaRTA, INSA, University of Barcelona, Barcelona, Spain; 19 Lipid Clinic, Department of Endocrinology and Nutrition, Institut d’Investigacions Biomediques August Pi Sunyer (IDIBAPS), Hospital Clinic, University of Barcelona, Barcelona, Spain; University of Granada, Spain

## Abstract

**Background:**

The traditional Mediterranean dietary pattern (MedDiet) is associated with longevity and low rates of cardiovascular disease (CVD). However, there is little information on who is more likely to follow this food pattern.

**Aim:**

To evaluate how different factors are associated with lower MedDiet adherence in older Spanish subjects.

**Methods:**

We included 7305 participants (men aged 55–80 y, women 60–80 y) at high-risk of CVD recruited into the PREDIMED trial (ISRCTN35739639). Socioeconomic, anthropometric, lifestyle characteristics and CVD risk factors were recorded. A validated 14-item questionnaire was used to evaluate MedDiet adherence at baseline. Multivariate models were used to estimate odds ratios (OR) and 95% confidence intervals for lower adherence to the MedDiet (<9 points out of 14) and ascertain factors independently associated with it.

**Results:**

Former smoking (OR = 0.87; 95% CI, 0.78–0.98), physical activity (OR for the 3^rd^ vs. the 1^st^tertile: 0.69; 0.62–0.78), and higher educational level (OR for university vs. less than primary school: 0.54; 0.38–0.77) were associated with higher MedDiet adherence. Conversely, having a larger waist-to-height ratio (OR for 0.1 units, 1.35; 1.22–1.49), being diabetic (OR = 1.13; 1.03–1.24), being single (OR = 1.27; 1.01–1.61) or divorced or separated (OR = 1.44; 1.09–1.89), and current smoking (OR = 1.28; 1.11–1.47) were associated with lower adherence.

**Conclusions:**

Participants with little education, a larger waist-to-height ratio, or diabetes and those who were less physically active, single, divorced or separated, or smokers were less likely to adhere to the MedDiet, an ideal model for food choices. Stronger efforts of health promotion are needed in these groups to foster adoption of the MedDiet.

## Introduction

There has been a growing interest in the association between diet and prevention of chronic diseases because foods and nutrients can act synergistically to increase or reduce mortality through chronic disease[1]. Researchers have investigated a variety of dietary patterns and their long-term health effects in populations. Studies have revealed the highly beneficial health effects of the Mediterranean diet (MedDiet) and its significant inverse relationship with cardiovascular disease (CVD), obesity, and cancer; therefore, it is commonly associated with lower mortality and higher longevity [2]–[9].

The MedDiet is a traditional food pattern present in the olive oil producing areas of the Mediterranean basin. The MedDiet is an ideal dietary model because it is plant-based, each food group is eaten in moderation, the foods are local and ecologically friendly, and the cultural lifestyle that accompanies this food pattern embodies a sense of community, physical activity, and adequate rest [10]. The primary components of this diet include an abundance of vegetables, fruits, legumes, nuts, and olive oil; a moderate intake of fish, dairy products, and wine; small portions of meat and poultry, and little consumption of sweets. A key characteristic of this diet is the low amount of animal and trans fat [11]. Virgin olive oil, the primary source of fat along with plant foods and nuts, makes the MedDiet ideal because these fresh foods undergo minimal processing so they are rich in fiber, antioxidants, and essential micro and macronutrients [11]–[13].

Although the MedDiet has been proposed as yielding long life expectancy, recent research has found that the Mediterranean populations that once exhibited these beneficial effects are abandoning the MedDiet and associated healthy lifestyles to adopt in its place unhealthy Westernized food patterns. In Spain, young people are reported to have given up the traditional MedDiet and are instead opting for less healthy choices [14]. As a result of the Mediterranean region’s assimilation to Western culture, their diet has drifted away from the traditional MedDiet and a new MedDiet pyramid has been designed to accommodate these changes [10]. There is a need to investigate which lifestyle factors are associated with adherence to the MedDiet among community-dwelling high-risk subjects and to apply this knowledge to educate the general population about health risks to reduce CVD rates in the Mediterranean regions.

The causal relationship linking the MedDiet with a lower risk of chronic disease appears to be sound. However, not much research has been performed to characterize who is more likely to adhere to the MedDiet. Our aim was to investigate which socioeconomic and lifestyle factors are associated with lower adherence to the MedDiet in the frame of the PREDIMED trial, a primary prevention feeding intervention study conducted in Spain in community-dwelling subjects at high risk for CVD.

## Methods

### Study Design/Participants

Data for this study stem from the PREDIMED (PREvención con DIeta MEDiterránea) Study, a clinical trial aimed to assess the effects of the MedDiet on the primary prevention of CVD (Controlled-trials.com number, ISRCTN35739639). A detailed description of the trial has been published elsewhere [12]. Briefly, eligible participants were men (55–80 years) and women (60–80 years) drawn from databases of primary care facilities in Spain, who were free of CVD at baseline and had either type-2 diabetes or at least 3 major cardiovascular risk factors [smoking, hypertension, high LDL-cholesterol, low HDL-cholesterol, high BMI, or family history of premature coronary heart disease (CHD)]. In the PREDIMED trial, 7447 participants were enrolled and randomly allocated to 3 intervention groups: a MedDiet supplemented with extra-virgin olive oil, a MedDiet supplemented with mixed nuts, or a control diet. The primary endpoint included cardiovascular death, myocardial infarction, or stroke. Of the 7447 participants who were enrolled, 7305 had available information for the present analyses. When baseline characteristics of participants who were included or excluded were compared, no significant differences were observed except for age (67.0 vs. 68.7) and diabetes (48% vs. 61%).

### Assessed Variables

A 33-item eligibility questionnaire and a 77-item general questionnaire were used to collect information on sociodemographic characteristics, educational level and medical conditions, among others [12]. Characteristics registered at baseline included age, sex, educational level, physical activity, diabetes, hypertension, smoking status, presence of high blood total cholesterol, family history of CHD, body mass index (BMI), waist circumference (WC), systolic and diastolic blood pressure, cholesterol levels (LDL and HDL), and alcohol intake. Smoking status was grouped in never smokers, current smokers, or former smokers. Educational level included university, secondary school, primary school, or less than primary school. Weight, height and WC were directly measured by registered nurses who had been previously trained and certified to implement the PREDIMED protocol and were hired to work full-time for this trial, as previously described [12]. BMI was calculated as weight (kg) divided by the square of height (m). Waist-to-height ratio was computed as the WC divided by height, both in centimeters [15], [16]. A validated 137-item food frequency questionnaire was used to assess the usual diet of each individual [17]. Food and nutrient intakes were calculated as frequency x nutrient composition of specified portion sizes, where frequencies were measured in 9 categories for each food item. Nutrient composition of foods was derived from Spanish food composition tables. Since wine consumption was an item included in the 14-item score of adherence to the traditional Mediterranean diet (see below), we derived non-wine alcohol consumption from sources other than wine. Non-wine alcohol consumption was split into categories of low (men: <10 g/d, women: <5 g/d), moderate (men: 10–50 g/d, women: 5–10 g/d), and high (men: ≥50 g/d, women: ≥10 g/d). Information on physical activity was collected with the validated Spanish version of the Minnesota Leisure-Time Physical Activity questionnaire [18], [19]. Taking into account the information on 67 different activities from this questionnaire and their typical average energy expenditure we derived total metabolic equivalents-minutes per day (METs-min/day) for each participant [18], [19]. Blood pressure, weight, and height were all measured by trained personnel [12].

In order to assess baseline adherence to the MedDiet, the participants were given a validated 14-item questionnaire that they were scored on [15], [20] (Table S1). The questions were based on number of servings and frequencies of consumption for typical foods or food groups of the MedDiet such as olive oil, nuts, fruits, wine, seafood, legumes, poultry; or questions about low consumption of foods that are not part of the traditional MedDiet, such as red or processed meats, sweetened beverages, and sweets, commercial bakery or sugary desserts. The questions had criteria for servings or frequencies that had to be met in order to earn a point. Each point earned corresponds to an increase in compliance with the MedDiet, so scores closer to 14 reflected high adherence and vice versa. This 14-item score was used both to assess changes in dietary habits during the trial and associations between different lifestyle variables and adherence to the MedDiet.

### Statistical Methods

The participants were grouped into either the low adherence group (<9 points) (n = 3390) or good adherence group (≥9 points) (n = 4013) based on their baseline score for the 14-item questionnaire. The independent association between each of the baseline characteristics and adherence to the MedDiet was assessed in two ways. First, logistic regression models were used to determine the crude and multivariate odds ratio (OR) to assess the association between the baseline participants’ characteristics and a low adherence to the MedDiet (<9 points). Ratios greater than 1 indicate low adherence compared to the reference and ratios less than 1 indicate good adherence compared to the reference. Second, mean scores were calculated for each baseline characteristic in order to compare the average levels of adherence to the MedDiet across categories of each independent predictor. A t-test or ANOVA was used to compare crude means. A multiple linear regression model was used to assess adjusted mean differences in MedDiet adherence between categories of lifestyle or risk factors variables. For physical activity and non-wine alcohol consumption, P-values for trend were calculated by assigning each category its median and treating the resulting variable as quantitative. Pre-specified interactions between several baseline characteristics and sex were also assessed using 1-degree of freedom likelihood ratio tests in logistic regression models for sex*age, sex*alcohol and sex*obesity indexes, all as continuous variables. Analyses stratified by sex are presented in Tables S2 and S3 because of a significant interaction between age and sex. All P-values presented are two-tailed and statistical significance was defined a priori at P <0.05. Data analyses were performed using SAS 9.3 (SAS Institute Inc, Cary NC).

## Results

The mean score in the 14-item questionnaire was 8.6 (SD: 1.9). The percentage of attaining one point was highest for consuming less than one serving of butter, margarine or cream per day (91%) and lowest for consuming at least three servings of legumes per week (27%). Other items with a percentage of scoring higher than 85% were using olive oil as main culinary fat (90%); consuming less than one sweetened and/or carbonated beverage per day; and consuming less than one serving of red meat, hamburger or meat products per day (87%).

Table 1 shows the baseline characteristics of the 7305 participants included in the study according to their baseline adherence to the MedDiet. In our study, men had a higher adherence to the MedDiet than women. Compared to participants with a lower adherence to the MedDiet, participants with higher adherence were less likely to be diabetics or current smokers, whereas more former smokers were in the good adherence group. A higher percentage of participants in the high adherence group engaged in physical activity and had university or secondary school education.

**Table 1:** Baseline characteristics of participants in the PREDIMED trial according to baseline adherence to the Mediterranean diet assessed with the 14-item PREDIMED brief questionnaire.

Characteristics at Baseline*	Low Adherence to MedDiet(<9 points)(n = 3349)	High Adherence to MedDiet(≥9 points)(n = 3956)
Age (years)	67.0 (6.2)	67.0 (6.2)
Sex (% women)	58.9	56.0
Smoking status		
Never (%)	62.4	60.3
Former (%)	22.2	26.9
Current (%)	15.4	12.8
Diabetes (%)	49.9	46.9
Family history of premature CHD (%)	21.5	23.3
BMI (kg/m^2^)	30.3 (3.8)	29.7 (3.8)
Waist circumference (cm)	101.5 (10.3)	99.6 (10.7)
Waist-to-height ratio	0.64 (.07)	0.62 (.07)
Hypertension (%)	83.6	82.2
Systolic blood pressure (mmHg)	148.9 (19.0)	148.4 (19.1)
Diastolic blood pressure (mmHg)	82.8 (10.2)	82.8 (10.2)
High total cholesterol (%)	72.5	72.3
HDL-cholesterol (mg/dL) ***	52.5 (12.8)	53.4 (12.8)
LDL-cholesterol (mg/dL)***	130.7 (33.3)	132.6 (34.1)
Physical activity (METS-min/day)	210.4 (221.8)	249.1 (252.6)
Educational Level		
University (%)	6.6	7.8
Secondary school (%)	15.1	16.0
Primary school (%)	75.1	74.4
Less than primary school (%)	3.3	1.8
Alcohol consumption from sources other than wine (g/day)**		
Low (%)	91.4	91.7
Moderate (%)	6.5	6.8
High (%)	2.1	1.5
Marital Status		
Married (%)	74.3	78.1
Single or Religious (%)	4.7	4
Widowed (%)	17.5	15.4
Divorced or Separated (%)	3.6	2.6

* : mean (SD) unless otherwise stated.

** low: <10 g/d (men), <5 g/d (women); moderate: 10–50 g/d (men)/5–10 g/d (women); high: ≥50 g/d (men)/≥10 g/d (women).

M: men; W: women.

*** low adherence n = 1,853, high adherence n = 2,471, total n = 4,324.

Table 2 displays the OR (95% confidence interval) for low adherence for each baseline characteristic. The baseline characteristics that were significantly associated with adherence to the MedDiet in the crude analysis included sex, smoking, BMI, waist circumference, waist-to-height ratio, physical activity, educational level, marital status, and diabetes. In the multivariable analysis, being a former smoker, higher physical activity, and higher educational level were associated with better adherence to the MedDiet, while smoking, a high waist-to-height ratio, being single, divorced and separated, and diabetes were associated with a lower adherence. We observed a statistically significant interaction between age and sex (P value for the interaction: 0.005) on adherence to the MedDiet. Since adherence was significantly different between age groups for both sexes, results are presented separately for men and women (Table S2). Concretely, older men adhered more to the traditional MedDiet than younger men, whereas no association between age and adherence to the MedDiet was observed among women. The direct association between age and adherence to the MedDiet among men remained significant after restricting age to ≥60 years and ≤70 years. Among female participants, civil status was no longer associated to adherence to the MedDiet.

**Table 2:** Odds ratios (95% confidence intervals) for low adherence (<9 points) to the Mediterranean diet in the PREDIMED trial according to baseline characteristics.

Characteristics at Baseline	Crude OR (95% CI)	P-value		Multivariate OR(95% CI)†	P-value	
Sex						
Men (n = 3165)	1 (ref.)					
Women (n = 4282)	1.12 (1.02–1.23)	0.01				
Age (years)						
<65 (n = 2832)	1 (ref.)					
≥65 (n = 4473)	0.97 (0.89–1.07)	0.58				
Diabetes						
no (n = 3778)	1 (ref.)			1 (ref.)		
yes (n = 3527)	1.13 (1.03–1.24)	0.01		1.13 (1.03–1.24)	0.01	
Hypertension						
no (n = 1255)	1 (ref.)					
yes (n = 6050)	1.10 (0.97–1.25)	0.12				
Smoking status						
Never(n = 4474)	1 (ref.)			1 (ref.)		
Former(n = 1809)	0.80(0.71–0.89)			0.87 (0.78–0.98)		
Current(n = 1022)	1.16 (1.02–1.33)	<0.001		1.28 (1.11–1.47)	0.001	
High total cholesterol						
no (n = 2017)	1(ref.)					
yes (n = 5288)	1.01 (0.91–1.12)	0.85				
Family history of premature CHD						
no (n = 5665)	1 (ref.)					
yes (n = 1640)	0.90 (0.81–1.01)	0.06				
BMI (per 1 kg/m^2^)	1.04 (1.02–1.05)	<0.001				
Waist circumference (per 5 cm)	1.09 (1.06–1.11)	<.0001				
Waist-to-height ratio (per 0.1 units)	1.38(1.29–1.49)	<.0001		1.35 (1.22–1.49)	<.0001	
Systolic blood pressure (per 5 mmHg)	1.01(0.99–1.02)	0.36				
Diastolic blood pressure (per 5 mmHg)	1.00(0.98–1.02)	0.87				
Physical activity* (METS-min/day)						
T1 (n = 2392)	1 (ref.)			1 (ref.)		
T2 (n = 2468)	0.85 (0.76–0.95)			0.87 (0.78–0.98)		
T3 (n = 2445)	0.65 (0.58–0.73)	<.0001		0.69 (0.62–0.78)	<0.001	
Educational Level						
Less than primary school (n = 180)	1 (ref.)		1 (ref.)			
Primary school (n = 5458)	0.56 (0.41–0.75)		0.60 (0.44–0.82)			
Secondary School (n = 1136)	0.52 (0.38–0.72)		0.58 (0.42–0.81)			
University (n = 531)	0.47 (0.33–0.66)	0.001	0.54 (0.38–0.77)		0.04	
Alcohol consumption from sources other than wine (g/day)**						
Low (n = 4945)	1 (ref.)					
Moderate (n = 1703)	0.97 (0.80–1.16)					
High (n = 799)	1.34(0.95–1.90)	0.72				
Marital Status						
Married (n = 5576)	1 (ref.)		1 (ref.)			
Single or Religious (n = 314)	1.23(0.98–1.54)		1.27(1.01–1.61)			
Widowed (n = 1195)	1.19(1.05–1.35)		1.11(0.98–1.26)			
Divorced or Separated (n = 220)	1.46(1.12–1.92)	0.001	1.44(1.09–1.89)		0.01	

* : T1: tertile 1 (<105 METS-min/day); T2: tertile 2 (≥105–<257.1 METS-min/day); T3: tertile 3 (≥257.1 METS-min/day).

** : low: <10 g/d (men), <5 g/d (women); moderate: 10–50 g/d (men)/5–10 g/d (women); high: ≥50 g/d (men)/≥10 g/d (women).

† : Adjusted for all other variables with a significant OR in the multivariable model.

Table 3 shows the mean scores of the 14-item questionnaire and the mean differences within each baseline characteristic. Participants who smoked, were obese, had a higher waist circumference or waist-to-height ratio, were single, divorced or separated, and had hypertension or diabetes had a lower score of adherence to the MedDiet, whereas more physically active or higher educated participants scored higher in the 14-item questionnaire in the multivariable model. The interaction between age and sex yielded a P value of 0.0001, a reason why the results were again separately calculated for men and women (Table S3). Older men (≥65 yrs) had a higher score of adherence to the MedDiet, while no association between age and adherence to the MedDiet was observed among women. In the stratified analyses, obesity, a high waist circumference, and hypertension were no longer significantly associated with the 14-item score.

**Table 3:** Mean scores and mean differences (95% CI) in the 14-item questionnaire of adherence to the Mediterranean diet. The PREDIMED trial.

Characteristics at Baseline	Mean(95% CI)	P-value	Mean Difference(95% CI)†	P-value
Sex				
Men (n = 3165)	8.77 (8.70 to 8.84)			
Women (n = 4282)	8.58 (8.53 to 8.64)	<0.001		
Age (years)				
<65 (n = 2832)	8.66 (8.59 to 8.73)			
≥65 (n = 4473)	8.67 (8.61 to 8.72)	0.88		
Diabetes				
no (n = 3778)	8.74 (8.68 to 8.80)		0 (ref.)	
yes (n = 3527)	8.58(8.52 to 8.64)	<0.001	−0.18 (−0.27 to −0.09)	<0.001
Hypertension				
no (n = 1255)	8.76 (8.66 to 8.87)		0 (ref.)	
yes (n = 6050)	8.64 (8.59 to 8.69)	0.04	−0.12 (−0.24 to −0.003)	0.04
Smoking status				
Never(n = 4474)	8.63 (8.57 to 8.68)		0 (ref.)	
Former(n = 1809)	8.83 (8.74 to 8.92)		0.07 (−0.04 to 0.17)	
Current(n = 1022)	8.52 (8.41 to 8.64)	<0.001	−0.26 (−0.39 to −0.13)	<0.001
High blood total cholesterol				
no (n = 2017)	8.66 (8.58 to 8.74)			
yes (n = 5288)	8.67 (8.61 to8.72)	0.90		
Family history of CHD				
no (n = 5665)	8.64 (8.59 to 8.69)			
yes (n = 1640)	8.75 (8.66 to 8.85)	0.02		
BMI (kg/m2)				
<30	8.82 (8.76 to 8.88)		0 (ref.)	
≥30	8.49 (8.42 to 8.55)	<.0001	−0.15 (−0.25 to −0.04)	0.01
Waist circumference (cm)				
<102 in M/<88 in W	8.97 (8.88 to 9.05)		0 (ref.)	
≥102 in M/> = 102 in W	8.55(8.50 to 8.60)	<.0001	−0.16 (−0.30 to −0.02)	0.02
Waist-to-height ratio				
<0.6	8.92 (8.84 to 8.99)		0 (ref.)	
≥0.6	8.54 (8.48 to 8.59)	<.0001	−0.13 (−0.27 to −0.003)	0.045
Physical activity (METS-min/day)*				
T1 (n = 2392)	8.47 (8.39 to 8.54)		0 (ref.)	
T2 (n = 2468)	8.62 (8.54 to 8.69)		0.11(0.001 to 0.22)	
T3 (n = 2445)	8.90 (8.83 to 8.98)	<.0001	0.34 (0.23 to 0.45)	<0.001
Educational Level				
Less than primary school (n = 180)	7.96 (7.68 to 8.24)		0 (ref.)	
Primary school (n = 5458)	8.64 (8.59 to 8.69)		0.59 (0.31 to 0.87)	
Secondary School (n = 1136)	8.79 (8.68 to 8.90)		0.70 (0.39 to 1.00)	
University (n = 531)	8.87 (8.71 to 9.03)	<0.001	0.75 (0.43 to 1.08)	<0.001
Alcohol consumption from sources other than wine (g/day)*				
Low (n = 4945)	8.66 (8.62 to 8.71)			
Moderate(n = 1703)	8.69(8.52 to 8.86)			
High (n = 799)	8.55 (8.22 to 8.88)	0.61		
Marital Status				
Married (n = 5576)	8.71 (8.37 to 8.77)		0 (ref.)	
Single or Religious (n = 314)	8.49 (8.28 to 8.70)		−0.28 (−0.50 to −0.07)	
Widowed (n = 1195)	8.53 (8.42 to 8.64)		−0.09 (−0.22 to 0.03)	
Divorced or Separated (n = 220)	8.29 (8.04 to 8.54)	<0.001	−0.40 (−0.65 to −0.14)	0.001

* : T1: tertile 1 (<105 METS-min/day); T2: tertile 2 (≥105–<257.1 METS-min/day); T3: tertile 3 (≥257.1 METS-min/day).

** : low: <10 g/d (men), <5 g/d (women); moderate: 10–50 g/d (men)/5–10 g/d (women); high: ≥50 g/d (men)/≥10 g/d (women).

† : Adjusted for all other variables with a significant association in the multivariable model.

M: men; W: women.

## Discussion

In this study we assessed how socioeconomic and lifestyle characteristics and the presence of risk factors for CVD influenced baseline adherence to the traditional MedDiet in a cohort of 7305 older Spanish subjects at high risk for CVD participating in the PREDIMED trial, a primary prevention nutritional intervention trial. The results suggest that former smokers and subjects with a higher level of physical activity, higher educational level and lower waist-to-height ratio tended to adhere more to the MedDiet, whereas current smokers, single or divorced participants, and those with diabetes were less likely to adhere to this dietary pattern.

Only among men, older subjects (≥65 yrs) were slightly more adherent to the MedDiet than men younger than 65 yrs. A possible explanation may be that older citizens have a more traditional lifestyle and are reluctant to eat outside of the diet they grew up with, while younger generations have greater exposure to new foods and are more open-minded to try new, more fashionable foods. Although our participants were all older than 55 years and the age range was narrow, other studies with broader age ranges have confirmed that there is an age difference in adherence to the traditional MedDiet in Spain, Greece and Cyprus [21]–[25]. It is interesting that the direct association between age and adherence to the MedDiet was significant within a narrow age range and it remained so even after restricting age to 60–70 years. Our results show that participants with diabetes had a lower score of adherence to the MedDiet than non-diabetic ones, suggesting that unhealthy dietary habits might be related in part to the diabetic status, as reported for other populations [26], [27]. It is well established that dietary habits have a profound influence on development of type-2 diabetes [28]. There is a consistent body of evidence signaling precisely the MedDiet as particularly suited for diabetes prevention, as shown by observational studies with outcomes on incident diabetes [29], [30] or glycemic control [31], [32] and the results of randomized feeding trials in high-risk subjects that have assessed changes in risk factors, including insulin resistance and inflammatory biomarkers [33], [34], incident diabetes [35], or the need for antihyperglycemic therapy in patients with new-onset diabetes [36]. Of note, three of these studies were conducted in PREDIMED cohorts [32], [34], [35]. Also, when compliance with the intervention was prospectively assessed among the first 1048 participants in the PREDIMED trial, improvement in adherence to the MedDiet among diabetic men in the intervention groups was poorer than among those without diabetes [37]. Thus, the MedDiet might be useful both for diabetes prevention and as an adjunctive treatment to improve diabetic control.

In our study, participants with a low waist-to-height ratio were more likely to be compliant with the MedDiet. This specific association might have important implications for public health and health promotion in the context of the generalized obesity pandemic and it has been dealt with in detail by our group in a separate paper [15]. Other prospective studies have also shown that a higher adherence to the traditional MedDiet may have beneficial effects on long-term weight changes [4], [5], [32]. The inverse association between adherence to the traditional MedDiet and weight gain could translate into public health policy promoting its adherence to tackle the current increasing levels of obesity in the Mediterranean area [38]–[41].

Our results also suggest that lower levels of physical activity are associated with poorer adherence to the MedDiet in both men and women. In addition, we found that smoking is associated with poorer adherence to the MedDiet. In past studies conducted in Greek and Spanish populations, current smokers were also less likely to adhere to the MedDiet than never smokers [22], [23], [42], [43]. More generally, the association between smoking and an overall poorer diet has been observed in other populations as well [44]. This is not surprising since current smokers have more negative health behaviors than former smokers and never smokers [45]. It has also been suggested that the diet of former smokers resembles more that of never smokers than the diet of current smokers [46], [47]. In our sample, the diet of former smokers was healthier than that of never smokers. This may be due to recent diagnosis of a chronic condition. That the two lifestyle factors of smoking and being physically inactive are independently associated with poorer adherence to the MedDiet concurs with previous studies that suggest clustering of unhealthy lifestyles [21], [24], [42]. This clustering of unhealthy lifestyle factors is informative for observational analyses since thorough adjustment for lifestyle variables is required to disentangle the effects of the departure of the traditional MedDiet on chronic diseases from those derived from lifestyle factors.

We found that less educated subjects tended to have a lower adherence to the MedDiet, which confirms the findings of previous research [22], [23]. Other studies have found either no association [21], [24] or an inverse association [42]. More research is needed in this area in order to accurately gauge this association. This is important because the educated population tends to play a model role and sets an example for the rest of society. If the educated population complies with the MedDiet, a large portion of the population may follow suit.

Our results also show that married men are more likely to comply with the MedDiet than those who are single, widowed, or divorced/separated. While the differences among the categories were highly significant for men, this was not the case for women. This may be related to the traditional role of older women in Spanish households, i.e., the responsibility of preparing meals. Previous research has suggested that married subjects of either sex are more likely to adhere to the MedDiet than those who are widowed, separated, or divorced [48].

Our study had some limitations. Like in any cross-sectional study, caution must be used when interpreting results due to the possibility of reverse causation bias. For example, it is likely that the inverse association of the MedDiet score with several measures of adiposity could set off a feedback loop, in that a better diet causes less fatness, which in turn reinforces healthy eating behaviors. Also, most data variables were self-reported, which poses potential subject bias. Calculations for food scores may have been biased due to misreporting by older subjects with poor memory or underreporting by those who were overweight or obese. There is a possibility of measurement error caused by subjects with high education intentionally or unintentionally reporting a healthier diet because they knew it was more socially acceptable. However, the 14-item score of adherence to the MedDiet has been previously validated in our study [20]. The cutoff point of 9 in the 14-item score may seem arbitrary, but it was chosen based on the results of a previous substudy of the PREDIMED trial, whereby scoring <9 was associated with clustering of cardiovascular risk factors [49]. The scores for the 14-item questionnaire may vary among participants since not all were recruited within the same time frame. Seasonal variability in availability of produce (fruits and vegetables) might cause inaccuracy in adherence scores. Nevertheless, this non-differential bias would have shifted our results towards the null. Our population was made of older high-risk subjects, thus the results may not be extrapolated to other populations. Nonetheless, they provide valuable information about a high-risk group that could particularly benefit from lifestyle intervention. A major strength of the study was the large sample size. Also, the 14-item MedDiet, the 137-item food frequency and the physical activity questionnaires were all comprehensive and had been previously validated.

Overall, our results suggest that, in order to improve health status, more effort is needed to inform about the MedDiet to individuals who smoke in spite of advice to the contrary; are diabetic or less educated; or have a greater waist perimeter. They also provide useful information for the design of future intervention studies regarding what type of participants would show optimal adherence to the MedDiet. The Mediterranean lifestyle, which encompasses diet and physical activity, needs to be reinforced in Mediterranean populations, especially to younger generations. Taking into account the beneficial health effects of the traditional MedDiet, there is an urgent need to enhance the population’s dietary habits. However, further research is necessary to confirm our findings.

## Supporting Information

Table S1 Short questionnaire to assess adherence to the Mediterranean diet. The PREDIMED trial 2003–2010.
(DOCX)

Table S2 Odds ratios (95% confidence intervals) for low adherence (<9 points) to the Mediterranean diet in the PREDIMED trial according to baseline characteristics.
(DOCX)

Table S3 Mean scores and mean differences (95% CI) in the 14-item questionnaire of adherence to the Mediterranean diet. The PREDIMED trial.
(DOCX)
