# Supplementary material for: Lifestyles and Risk Factors Associated with Adherence to the Mediterranean Diet: A Baseline Assessment of the PREDIMED Trial
Source: PLoS One. 2013 Apr 29;8(4):e60166. doi: 10.1371/journal.pone.0060166 (PMC3639284; doi:10.1371/journal.pone.0060166)
Supplement: Table S1 — Short questionnaire to assess adherence to the Mediterranean diet. The PREDIMED trial 2003–2010. (DOCX) [file pone.0060166.s001.docx]

**Table S1. Short questionnaire to assess adherence to the Mediterranean diet. The PREDIMED trial 2003-2010.**

| Questions | Criteria for 1 point |
| --- | --- |
| 1. Do you use olive oil as main culinary fat? | Yes |
| 1. How much olive oil do you consume in a given day (including oil used for frying, salads, out-of-house meals, etc.)? | ≥ 4 tbsp/d |
| 1. How many vegetable servings do you consume per day? [1 serving: 200 g (consider side dishes as half a serving)] | ≥ 2 (≥ 1 portion raw or as a salad) |
| 1. How many fruits units (including natural fruit juices) do you consume per day? | ≥ 3 serv./d |
| 1. How many servings of red meat, hamburger or meat products (ham, sausage, etc.) do you consume per day? | < 1 |
| 1. How many servings of butter, margarine, or cream do you consume per day? (1 serving: 12 g) | <1 |
| 1. How many sweetened and/or carbonated beverages do you drink per day? | <1 |
| 1. How much wine do you drink per week? | ≥ 7 glasses |
| 1. How many servings of legumes do you consume per week? (1 serving: 150 g) | ≥ 3 |
| 1. How many servings of fish or shellfish do you consume per week? (1 serving 100-150g g of fish or 4-5 units or 200 g of shellfish) | ≥ 3 |
| 1. How many times per week do you consume commercial sweets or pastries (not homemade), such as cakes, cookies, biscuits or custard? | < 2 |
| 1. How many servings of nuts (including peanuts) do you consume per week? (1 serving 30 g) | ≥ 3 |
| 1. Do you preferentially consume chicken, turkey or rabbit meat instead of veal, pork, hamburger or sausage? | Yes |
| 1. How many times per week do you consume vegetables, pasta, rice or other dishes seasoned with sofrito (sauce made with tomato and onion, leek or garlic and simmered with olive oil)? | ≥ 2 |
