# Supplementary material for: Lifestyles and Risk Factors Associated with Adherence to the Mediterranean Diet: A Baseline Assessment of the PREDIMED Trial
Source: PLoS One. 2013 Apr 29;8(4):e60166. doi: 10.1371/journal.pone.0060166 (PMC3639284; doi:10.1371/journal.pone.0060166)
Supplement: Table S2 — Odds ratios (95% confidence intervals) for low adherence (<9 points) to the Mediterranean diet in the PREDIMED trial according to baseline characteristics. (DOCX) [file pone.0060166.s002.docx]

**Table S2. Odds ratios (95% confidence intervals) for low adherence (<9 points) to the Mediterranean diet in the PREDIMED trial according to baseline characteristics.**

|  | Men | | | |  | Women | | | |
| --- | --- | --- | --- | --- | --- | --- | --- | --- | --- |
| Characteristics at Baseline | Crude OR  (95% CI) | p-value | Multivariate OR  (95% CI)† | p-value |  | Crude OR  (95% CI) | p-value | Multivariate OR  (95% CI) † | p-value |
| Age (years) |  |  |  |  |  |  |  |  |  |
| <65 (n=2832) | 1  (ref.) |  | 1  (ref.) |  |  | 1  (ref.) |  | 1  (ref.) |  |
| ≥65 (n=4473) | 0.83 (0.72-0.96) | 0.01 | 0.81  (0.70-0.94) | 0.01 |  | 1.08 (0.95-1.23) | 0.22 | 1.02  (0.89-1.17) | 0.79 |
| Diabetes |  |  |  |  |  |  |  |  |  |
| no (n=3778) | 1  (ref.) |  | 1  (ref.) |  |  | 1  (ref.) |  | 1  (ref.) |  |
| yes (n=3527) | 1.04  (0.91-1.20) | 0.56 | 1.05  (0.90-1.21) | 0.55 |  | 1.22 (1.08-1.38) | 0.001 | 1.19  (1.05-1.35) | 0.01 |
| Hypertension |  |  |  |  |  |  |  |  |  |
| no (n=1255) | 1 (ref.) |  |  |  |  | 1  (ref.) |  |  |  |
| yes (n=6050) | 1.13  (0.95-1.34) | 0.18 |  |  |  | 1.05 (0.88-1.25) | 0.61 |  |  |
| Smoking status |  |  |  |  |  |  |  |  |  |
| Never  (n=4474) | 1  (ref.) |  | 1  (ref.) |  |  | 1  (ref.) |  | 1  (ref.) |  |
| Former  (n=1809) | 0.92  (0.77-1.09) |  | 0.89  (0.75-1.06) |  |  | 0.74  (0.59-0.94) |  | 0.74  (0.58-0.95) |  |
| Current  (n=1022) | 1.33  (1.09-1.62) | 0.001 | 1.26  (1.03-1.54) | 0.001 |  | 1.14  (0.87-1.48) | 0.03 | 1.22  (0.93-1.61) | 0.01 |

**Table S2 (continued).Odds ratios (95% confidence intervals) for low adherence (<9 points) to the Mediterranean diet in the PREDIMED trial according to baseline characteristics.**

|  | Men | | | |  | Women | | | |
| --- | --- | --- | --- | --- | --- | --- | --- | --- | --- |
| Characteristics at Baseline | Crude OR  (95% CI) | p-value | Multivariate OR  (95% CI) † | p-value |  | Crude OR  (95% CI) | p-value | Multivariate OR  (95% CI) † | p-value |
| High total cholesterol |  |  |  |  |  |  |  |  |  |
| no (n=2017) | 1  (ref.) |  |  |  |  | 1  (ref.) |  |  |  |
| yes (n=5288) | 1.13  (0.97-1.31) | 0.12 |  |  |  | 0.89  (0.77-1.03) | 0.11 |  |  |
| Family history of premature CHD | |  |  |  |  |  |  |  |  |
| no (n=5665) | 1  (ref.) |  |  |  |  | 1  (ref.) |  |  |  |
| yes (n=1640) | 0.86  (0.71-1.03) | 0.11 |  |  |  | 0.90  (0.79-1.04) | 0.15 |  |  |
| BMI (per 1 kg/m^2^) | 1.04  (1.01-1.06) | .001 |  |  |  | 1.04  (1.02-1.05) | <.0001 |  |  |
| Waist circumference (per 5 cm) | 1.11  (1.06-1.15) | <.0001 |  |  |  | 1.10  (1.07-1.13) | <.0001 |  |  |
| Waist-to-height ratio (per 0.1 units) | 1.40  (1.24-1.59) | <.0001 | 1.37  (1.18-1.59) | <.0001 |  | 1.36  (1.25-1.49) | <.0001 | 1.34  (1.17-1.54) | <.0001 |
| Systolic blood pressure (per 5 mmHg) | 1.01  (0.99-1.03) | 0.21 |  |  |  | 1.00  (0.99-1.02) | 0.74 |  |  |
| Diastolic blood pressure (per 5 mmHg) | 0.99  (0.96-1.02) | 0.58 |  |  |  | 1.01  (0.98-1.04) | 0.59 |  |  |

**Table S2 (continued).Odds ratios (95% confidence intervals) for low adherence (<9 points) to the Mediterranean diet in the PREDIMED trial according to baseline characteristics.**

|  | Men | | | |  | Women | | | |
| --- | --- | --- | --- | --- | --- | --- | --- | --- | --- |
| Characteristics at Baseline | Crude OR  (95% CI) | p-value | Multivariate OR  (95% CI) † | p-value |  | Crude OR  (95% CI) | p-value | Multivariate OR  (95% CI) † | p-value |
| Physical activity* (METS-min/day) |  |  |  |  |  |  |  |  |  |
| T1 (n=2392) | 1  (ref.) |  | 1  (ref.) |  |  | 1  (ref.) |  | 1  (ref.) |  |
| T2 (n=2468) | 0.97  (0.80-1.18) |  | 1.01  (0.83-1.23) |  |  | 0.79  (0.69-0.91) |  | 0.81  (0.70-0.93) |  |
| T3 (n=2445) | 0.72  (0.60-0.86) | <.0001 | 0.77  (0.64-0.92) | 0.001 |  | 0.63  (0.53-0.73) | <.0001 | 0.65  (0.55-0.76) | <.0001 |
| Educational Level |  |  |  |  |  |  |  |  |  |
| Less than primary school (n=180) | 1  (ref.) |  | 1  (ref.) |  |  | 1 (ref.) |  | 1 (ref.) |  |
| Primary school (n=5458) | 0.38  (0.14-0.99) |  | 0.34  (0.13-0.92) |  |  | 0.59  (0.43-0.82) |  | 0.64  (0.46-0.88) |  |
| Secondary School (n=1136) | 0.34  (0.13-0.89) |  | 0.29  (0.11-0.78) |  |  | 0.62  (0.43-0.90) |  | 0.71  (0.48-1.03) |  |
| University (n=531) | 0.34  (0.13-0.93) | 0.13 | 0.30  (0.11-0.83) | 0.03 |  | 0.45  (0.29-0.70) | 0.02 | 0.52  (0.33-0.82) | 0.02 |

**Table S2 (continued).Odds ratios (95% confidence intervals) for low adherence (<9 points) to the Mediterranean diet in the PREDIMED trial according to baseline characteristics.**

|  | Men | | | |  | Women | | | |
| --- | --- | --- | --- | --- | --- | --- | --- | --- | --- |
| Characteristics at Baseline | Crude OR  (95% CI) | p-value | Multivariate OR  (95% CI) † | p-value |  | Crude OR  (95% CI) | p-value | Multivariate OR  (95% CI) † | p-value |
| Alcohol Consumption from sources other than wine (g/day)** |  |  |  |  |  |  |  |  |  |
| Low (n=4945) | 1  (ref.) |  |  |  |  | 1  (ref.) |  |  |  |
| Moderate(n=1703) | 1.04  (0.84-1.27) |  |  |  |  | 0.96  (0.55-1.68) |  |  |  |
| High (n=799) | 1.34  (0.72-2.48) | 0.37 |  |  |  | 1.32  (0.87-2.01) | 0.29 |  |  |
| Marital Status |  |  |  |  |  |  |  |  |  |
| Married (n=5576) | 1  (ref.) |  | 1  (ref.) |  |  | 1  (ref.) |  | 1  (ref.) |  |
| Single or Religious (n=314) | 1.30  (0.92-1.83) |  | 1.27  (0.90-1.81) |  |  | 1.16  (0.86-1.57) |  | 1.31  (0.96-1.78) |  |
| Widowed (n=1195) | 1.47  (1.00-2.14) |  | 1.50  (1.02-2.21) |  |  | 1.11  (0.97-1.28) |  | 1.09  (0.94-1.26) |  |
| Divorced or Separated (n=220) | 1.76  (1.13-2.72) | 0.01 | 1.62  (1.04-2.53) | 0.02 |  | 1.28  (0.91-1.81) | 0.24 | 1.34  (0.94-1.90) | 0.13 |

**Table S2 (continued).Odds ratios (95% confidence intervals) for low adherence (<9 points) to the Mediterranean diet in the PREDIMED trial according to baseline characteristics.**

*: T1: tertile 1 (<105 METS-min/day); T2: tertile 2 (≥105-<257.1 METS-min/day); T3: tertile 3 (≥257.1 METS-min/day)

**: low: <10 g/d (men), <5 g/d (women); moderate: 10-50 g/d (men) / 5-10 g/d (women); high: ≥50 g/d (men) / ≥10 g/d (women)

†: Adjusted for all other variables with a significant OR in the multivariable model.

M: men; W: women
