# Supplementary material for: Lifestyles and Risk Factors Associated with Adherence to the Mediterranean Diet: A Baseline Assessment of the PREDIMED Trial
Source: PLoS One. 2013 Apr 29;8(4):e60166. doi: 10.1371/journal.pone.0060166 (PMC3639284; doi:10.1371/journal.pone.0060166)
Supplement: Table S3 — Mean scores and mean differences (95% CI) in the 14-item questionnaire of adherence to the Mediterranean diet. The PREDIMED trial. (DOCX) [file pone.0060166.s003.docx]

**Table S3. Mean scores and mean differences (95% CI) in the 14-item questionnaire of adherence to the Mediterranean diet. The PREDIMED trial**

|  | Men | | | |  | Women | | | |
| --- | --- | --- | --- | --- | --- | --- | --- | --- | --- |
| Characteristics at Baseline | Mean  (95% CI) | p-value | Mean Difference  (95% CI)† | p-value |  | Mean  (95% CI) | p-value | Mean Difference  (95% CI)† | p-value |
| Age (years) |  |  |  |  |  |  |  |  |  |
| <65 (n=2832) | 8.65  (8.54 to 8.75) |  | 0  (ref.) |  |  | 8.67  (8.57 to 8.77) |  | 0  (ref.) |  |
| ≥65 (n=4473) | 8.87  (8.78 to 8.96) | 0.001 | 0.26  (0.11 to 0.40) | .0004 |  | 8.54  (8.47 to 8.61) | 0.03 | -0.07  (-0.20 to 0.05) | 0.27 |
| Diabetes |  |  |  |  |  |  |  |  |  |
| no (n=3778) | 8.86  (8.76 to 8.96) |  | 0  (ref.) |  |  | 8.67  (8.59 to 8.74) |  | 0  (ref.) |  |
| yes (n=3527) | 8.70  (8.60 to 8.79) | 0.02 | -0.17  (-0.31 to -0.03) | 0.01 |  | 8.48  (8.39 to 8.56) | 0.002 | -0.15  (-0.27 to -0.03) | 0.01 |
| Hypertension |  |  |  |  |  |  |  |  |  |
| no (n=1255) | 8.82  (8.67 to 8.96) |  |  |  |  | 8.70  (8.54 to 8.86) |  |  |  |
| yes (n=6050) | 8.76  (8.68 to 8.84) | 0.48 |  |  |  | 8.56  (8.50 to 8.63) | 0.11 |  |  |
| Smoking status |  |  |  |  |  |  |  |  |  |
| Never  (n=4474) | 8.85 (8.72 to 8.99) |  | 0  (ref.) |  |  | 8.58  (8.52 to 8.64) |  | 0  (ref.) |  |
| Former  (n=1809) | 8.84  (8.74 to 8.94) |  | 0.01  (-0.15 to 0.17) |  |  | 8.79  (8.58 to 9.00) |  | 0.18  (-0.05 to 0.40) |  |
| Current  (n=1022) | 8.56  (8.43 to 8.70) | 0.002 | -0.24  (-0.43 to -0.05) | 0.01 |  | 8.40  (8.15 to 8.64) | 0.05 | -0.29  (-0.54 to -0.03) | 0.02 |

**Table S3 (continued).Mean scores and mean differences (95% CI) in the 14-item questionnaire of adherence to the Mediterranean diet. The PREDIMED trial**

|  | Men | | | |  | Women | | | |
| --- | --- | --- | --- | --- | --- | --- | --- | --- | --- |
| Characteristics at Baseline | Mean  (95% CI) | p-value | Mean Difference  (95% CI)† | p-value |  | Mean  (95% CI) | p-value | Mean Difference  (95% CI)† | p-value |
| High blood total cholesterol |  |  |  |  |  |  |  |  |  |
| no (n=2017) | 8.80  (8.68 to 8.92) |  |  |  |  | 8.51  (8.39 to 8.62) |  |  |  |
| yes (n=5288) | 8.76  (8.67to8.84) | 0.56 |  |  |  | 8.61  (8.54 to 8.67) | 0.16 |  |  |
| Family history of CHD |  |  |  |  |  |  |  |  |  |
| no (n=5665) | 8.74  (8.66 to 8.81) |  |  |  |  | 8.55 (8.49 to 8.62) |  |  |  |
| yes (n=1640) | 8.94 (8.78 to 9.11) | 0.02 |  |  |  | 8.67  (8.55 to 8.78) | 0.09 |  |  |
| BMI (kg/m2) |  |  |  |  |  |  |  |  |  |
| <30 | 8.89 (8.81 to 8.98) |  |  |  |  | 8.76  (8.67 to 8.84) |  |  |  |
| ≥30 | 8.60  (8.48 to 8.71) | <.0001 |  |  |  | 8.42  (8.34 to 8.50) | <.0001 |  |  |

**Table S3 (continued). Mean scores and mean differences (95% CI) in the 14-item questionnaire of adherence to the Mediterranean diet. The PREDIMED trial**

|  | Men | | | |  | Women | | | |
| --- | --- | --- | --- | --- | --- | --- | --- | --- | --- |
| Characteristics at Baseline | Mean  (95% CI) | p-value | Mean Difference  (95% CI)† | p-value |  | Mean  (95% CI) | p-value | Mean Difference  (95% CI)† | p-value |
| Waist circumference (cm) |  |  |  |  |  |  |  |  |  |
| <102 (88) | 8.96  (8.86 to 9.06) |  |  |  |  | 8.98  (8.83 to 9.13) |  | <102 (88) |  |
| ≥102 (88) | 8.63 (8.54 to 8.72) | <.0001 |  |  |  | 8.52  (8.45 to 8.58) | <.0001 | ≥102 (88) |  |
| Waist-to-height ratio |  |  |  |  |  |  |  |  |  |
| <0.6 | 8.97  (8.86 to 9.08) |  | 0  (ref.) |  |  | 8.87  (8.76 to 8.98) |  | 0  (ref.) |  |
| ≥0.6 | 8.65  (8.56 to 8.74) | <.0001 | -0.30  (-0.45 to -0.16) | <.0001 |  | 8.46 (8.40 to 8.53) | <.0001 | -0.34  (-0.47 to -0.21) | <.0001 |
| Physical activity (METS-min/day)* |  |  |  |  |  |  |  |  |  |
| T1 (n=2392) | 8.63  (8.49 to 8.77) |  | 0  (ref.) |  |  | 8.40 (8.31 to 8.49) |  | 0  (ref.) |  |
| T2 (n=2468) | 8.63  (8.50 to 8.75) |  | -0.05 (-0.24 to 0.14) |  |  | 8.61  (8.52 to 8.71) |  | 0.19  (0.06 to 0.32) |  |
| T3 (n=2445) | 8.94  (8.84 to 9.04) | <.0001 | 0.24  (0.06 to 0.41) | .001 |  | 8.85 (8.73 to 8.97) | <.0001 | 0.41  (0.26 to 0.56) | <.0001 |

**Table S3 (continued). Mean scores and mean differences (95% CI) in the 14-item questionnaire of adherence to the Mediterranean diet. The PREDIMED trial**

|  | Men | | | |  | Women | | | |
| --- | --- | --- | --- | --- | --- | --- | --- | --- | --- |
| Characteristics at Baseline | Mean  (95% CI) | p-value | Mean Difference  (95% CI)† | p-value |  | Mean  (95% CI) | p-value | Mean Difference  (95% CI)† | p-value |
| Educational Level |  |  |  |  |  |  |  |  |  |
| Less than primary school (n=180) | 8.05  (7.18 to 8.93) |  | 0  (ref.) |  |  | 7.95  (7.66 to 8.24) |  | 0  (ref.) |  |
| Primary school (n=5458) | 8.73  (8.64 to 8.81) |  | 0.73  (-0.14 to 1.60) |  |  | 8.59  (8.53 to 8.65) |  | 0.56  (0.26 to 0.86) |  |
| Secondary School (n=1136) | 8.87  (8.72 to 9.02) |  | .93  (0.05 to 1.81) |  |  | 8.65 (8.48 to 8.83) |  | 0.57  (0.23 to 0.92) |  |
| University (n=531) | 8.87  (8.67 to 9.07) | 0.09 | 0.91  (0.02 to 1.80) | 0.02 |  | 8.88  (8.59 to 9.16) | <.0001 | 0.77  (0.36 to 1.19) | 0.001 |
| Alcohol consumption from sources other than wine (g/day)* |  |  |  |  |  |  |  |  |  |
| Low (n=4945) | 8.78 (8.71 to 8.86) |  |  |  |  | 8.59 (8.53 to 8.65) |  |  |  |
| Moderate(n=1703) | 8.71 (8.53 to 8.90) |  |  |  |  | 8.44  (7.91 to 8.97) |  |  |  |
| High (n=799) | 8.73  (8.14 to 9.33) | 0.58 |  |  |  | 8.47  (8.08 to 8.87) | 0.45 |  |  |

**Table S3 (continued).Mean scores and mean differences (95% CI) in the 14-item questionnaire of adherence to the Mediterranean diet. The PREDIMED trial**

|  | Men | | | |  | Women | | | |
| --- | --- | --- | --- | --- | --- | --- | --- | --- | --- |
| Characteristics at Baseline | Mean  (95% CI) | p-value | Mean Difference  (95% CI)† | p-value |  | Mean  (95% CI) | p-value | Mean Difference  (95% CI)† | p-value |
| Marital Status |  |  |  |  |  |  |  |  |  |
| Married (n=5576) | 8.81  (8.72 to 8.90) |  | 0  (ref.) |  |  | 8.63  (8.54 to 8.71) |  | 0  (ref.) |  |
| Single or Religious (n=314) | 8.53  (8.11 to 8.94) |  | -0.26  (-0.59 to 0.07) |  |  | 8.46  (8.10 to 8.81) |  | -0.28  (-0.57 to 0.01) |  |
| Widowed (n=1195) | 8.60  (8.14 to 9.06) |  | -0.21  (-0.58 to 0.15) |  |  | 8.52  (8.38 to 8.66) |  | -0.07  (-0.20 to 0.07) |  |
| Divorced or Separated (n=220) | 8.14 (7.61 to 8.67) | 0.005 | -0.58  (-1.00 to -0.16) | 0.02 |  | 8.38  (7.98 to 8.79) | 0.18 | -0.28  (-0.61 to 0.04) | 0.09 |

*: T1: tertile 1 (<105 METS-min/day); T2: tertile 2 (≥105-<257.1 METS-min/day); T3: tertile 3 (≥257.1 METS-min/day)

**: low: <10 g/d (men), <5 g/d (women); moderate: 10-50 g/d (men) / 5-10 g/d (women); high: ≥50 g/d (men) / ≥10 g/d (women)

†: Adjusted for all other variables with a significant association in the multivariable model.
